# Initiators for Continuous Activator Regeneration (ICAR) Depolymerization

**DOI:** 10.1021/jacs.4c13785

**Published:** 2024-12-12

**Authors:** Glen R. Jones, Maria-Nefeli Antonopoulou, Nghia P. Truong, Athina Anastasaki

**Affiliations:** Laboratory for Polymeric Materials, Department of Materials, ETH Zürich, Vladimir-Prelog-Weg 5, 8093 Zürich, Switzerland

## Abstract

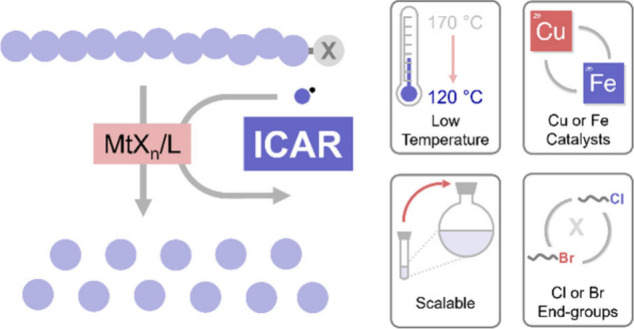

Chemical recycling of polymers synthesized by atom transfer radical polymerization (ATRP) typically requires high temperatures (i.e., 170 °C) to operate effectively, not only consuming unnecessary energy but also compromising depolymerization yields due to unavoidable end-group deterioration. To overcome this, the concept of initiators for continuous activator regeneration (ICAR) depolymerization is introduced herein as a broadly applicable approach to significantly reduce reaction temperatures for ATRP depolymerizations. Addition of commercially available free radical initiators enables the on-demand increase of depolymerization efficiency from <1% to 96%, achieving monomer generation at 120 °C, with conversions on par with thermal reversible addition–fragmentation chain transfer (RAFT) depolymerizations. Incubation studies confirm the elimination of deleterious side reactions at the milder temperatures employed, while the methodology can be scaled up to 1 g. The robustness and versatility of ICAR depolymerization is further demonstrated by the possibility to effectively depolymerize both chlorine and bromine terminated polymers and its compatibility with both copper and iron catalysts.

Reversible deactivation radical polymerizations (RDRPs) are invaluable tools for the synthesis of functional, well-defined macromolecules with complex architectures, achieving control over polymer chain growth by establishing an equilibrium between active and dormant species.^1−6^ In the case of atom transfer radical polymerization (ATRP), one of the most common RDRP techniques, this equilibrium arises from reversible “capping” of growing chains facilitated by a transition metal catalyst to yield polymeric materials with a halide chain-end.^7,8^ In recent years, materials synthesized by RDRP have also begun to attract considerable interest for their propensity to depolymerize at temperatures much lower than those required for polymers prepared via conventional radical polymerization.^9−12^ Reactivation of the functional chain-ends inherent in RDRPs can form radicals to give depolymerization closer to the ceiling temperature (*T*_c_) of the monomer,^13−19^ whereas conventional polymers require much higher temperatures to achieve chain scission to form radicals capable of depropagating.^20^

Depolymerization of ATRP-synthesized polymers is typically achieved by removing the halide chain-end with a transition metal catalyst, yielding a polymeric radical, which depropagates under thermodynamically favorable conditions. This concept was pioneered by Ouchi and co-workers in 2019, where a ruthenium complex was shown to catalyze the depolymerization of chlorine terminated polymethacrylates, albeit with relatively low conversions due to side reactions.^21^ Matyjaszewski and co-workers have developed depolymerization systems based on Cu^II^Cl_2_ and tris(2-pyridylmethyl)amine (TPMA) to achieve high monomer yields (67–76%) from chlorine-capped polymethacrylates at 170 °C (Figure 1A).^22,23^ These reactions proceed via an activator regenerated by the electron transfer (ARGET) ATRP mechanism, where TPMA acts as a reducing agent to continuously generate Cu^I^ activating species. UV/vis spectroscopy measurements revealed that reduction was not feasible at lower temperatures (i.e., 130 °C),^22^ necessitating the 170 °C conditions, 50 °C higher than that typically used in RAFT depolymerizations.^18^ Loss of end-group via a lactonization reaction can also be problematic at these elevated temperatures, leading to “dead” polymer chains incapable of being reactivated.^24^ This is particularly severe for bromine terminated polymers.^25^ A recent approach has enabled lower temperature depolymerization of chlorine-terminated polymethacrylates by using an iron catalyst in the presence of blue light (Figure 1B).^26^ Depolymerization was achieved at lower temperatures; however it required the combined use of a photoreactor and a heat source, presenting potential issues at higher scales,^27^ and employed an air sensitive Fe^II^ catalyst. A scalable and versatile depolymerization methodology for ATRP-synthesized polymers at low temperatures has not yet been realized.

**Figure 1:**
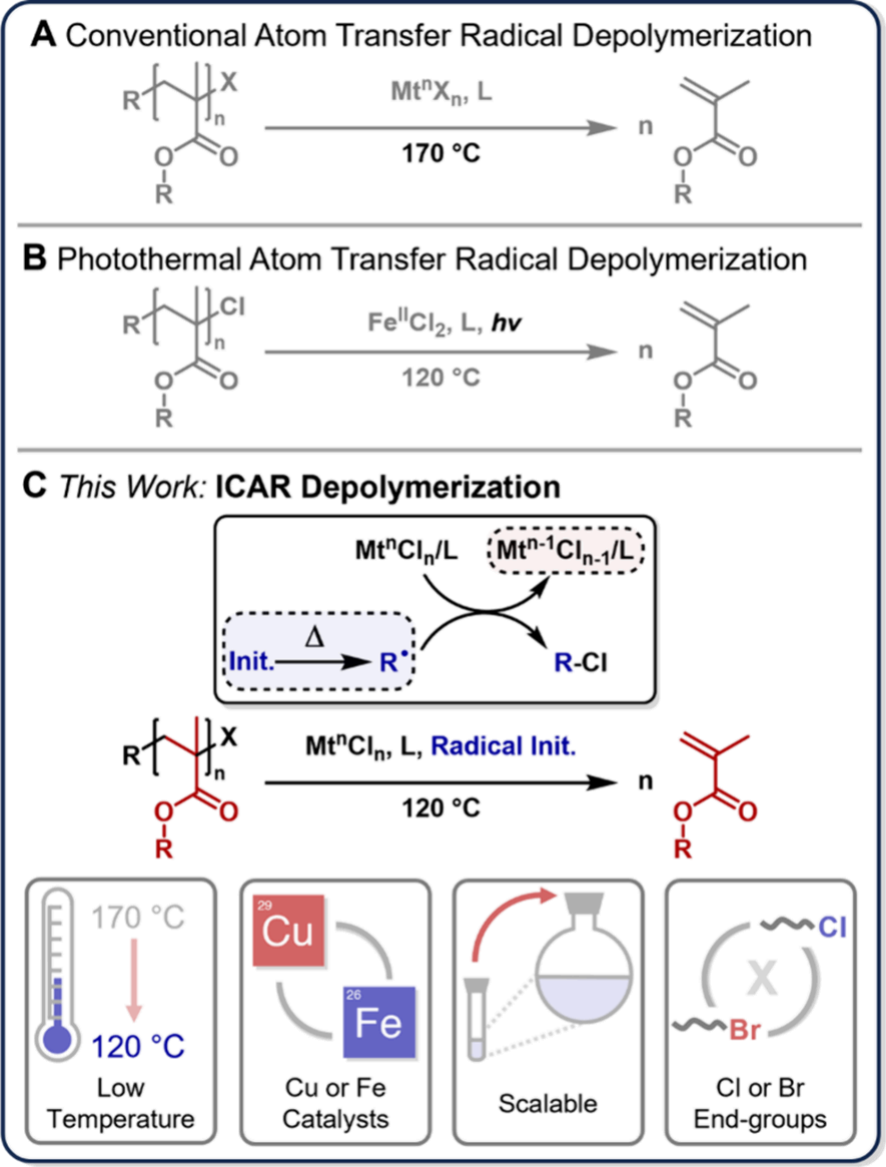
(A) Conventional atom transfer radical depolymerization. (B) Photothermal atom transfer radical depolymerization. (C) ICAR atom transfer radical depolymerization and key highlights.

Initiators for continuous activator regeneration (ICAR) ATRP works on the principle that a constant source of free radicals is able to (re)generate activator (i.e., Cu^I^) throughout a reaction, replenishing any that is consumed in unavoidable termination reactions.^28^ We were interested in whether this concept could also be applied to depolymerization of polymers synthesized by ATRP at relatively low temperatures (Figure 2A); reasoning that activator generation mediated by the decomposition of radical initiators could potentially allow for reduction at significantly lower reaction temperatures, while minimizing side reactions and avoiding the direct use of air sensitive Cu^I^ reagents.^29^ Herein we introduce ICAR depolymerization (Figure 1C) and show that simply adding an inexpensive free radical initiator allows for efficient depolymerization of ATRP-synthesized polymethacrylates at just 120 °C. Minimization of side reactions at this temperature allows polymers to remain stable until chain-end activation to induce “on-demand” depolymerization. Importantly, our approach is scalable and applicable to both chlorine and bromine terminated polymers in the presence of either copper or iron catalysts.

**Figure 2:**
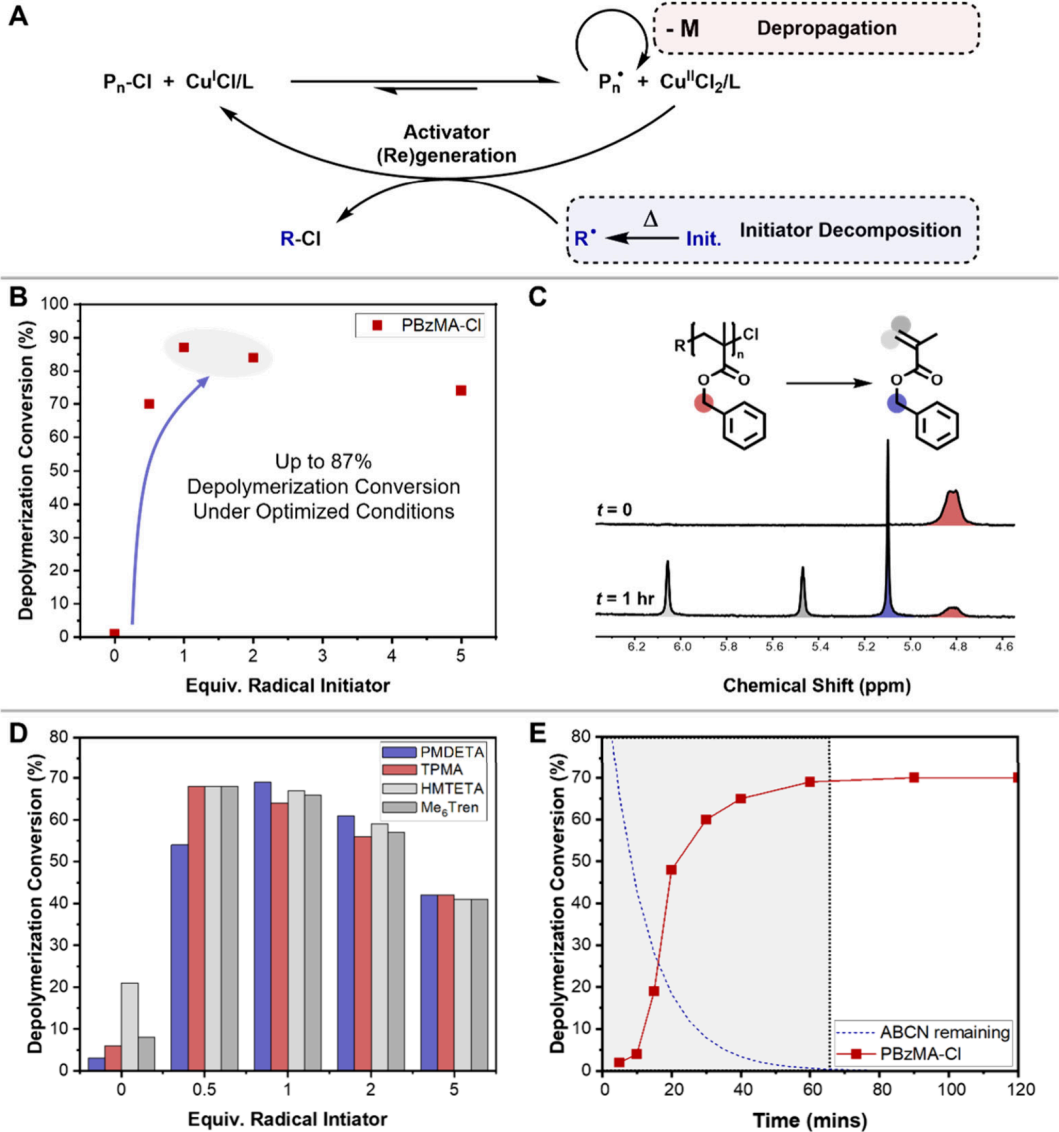
(A) Proposed mechanism of ICAR depolymerization. (B) Optimization of the depolymerization of PBzMA (5 mM RUC) with Cu^II^Cl_2_/TPMA and ABCN. (C) Representative ^1^H NMR showing the appearance of characteristic monomer peaks after depolymerization. (D) Ligand scope of ICAR depolymerization for PBzMA (50 mM RUC) with Cu^II^Cl_2_. (E) Kinetics of depolymerization (PBzMA, 50 mM, Cu^II^Cl_2_/PMDETA, 1 equiv of ABCN).

Benzyl methacrylate (BzMA) was first polymerized by ARGET ATRP, and purified to yield well-defined poly(benzyl methacrylate) (PBzMA, *Đ* = 1.13, Table S2, Figures S1–2) with a chlorine end-group. Depolymerization (5 mM repeat unit concentration (RUC)) was attempted at 120 °C with a typical ATRP depolymerization catalyst (Cu^II^Cl_2_/TPMA), yielding <1% monomer after 2 h. This low conversion is attributed to the inability of the ligand to reduce Cu^II^ to Cu^I^ at 120 °C.^22^ The experiment was then repeated with gradually increasing amounts of a radical initiator; 1,1′-azobis(cyclohexanecarbonitrile) (ABCN), selected due to its relatively high 10 h half-life (∼88 °C) making it suitable for reactions at 120 °C.^30^ Notably, ABCN addition drastically increases depolymerization conversion (Figure 2B), with 1 equiv with respect to polymer end-group yielding up to 87% monomer (96% depolymerization efficiency based on end-group fidelity, Figure S3), in stark contrast to initiator-free conditions. Figure 2C shows a typical ^1^H NMR spectrum of a reaction before and after depolymerization, with the appearance of characteristic vinyl peaks (gray) and a methylene peak at ∼5.1 ppm (blue) after 1 h indicating the presence of BzMA monomer. Importantly, control reactions where initiator was employed in the absence of ATRP catalyst yielded no appreciable depolymerization (Table S3), precluding the possibility of radical initiator directly activating chain-ends, thus it is likely that radicals act to generate Cu^I^ species from Cu^II^, as in ICAR ATRP.^28^ High amounts of initiator (e.g., 5 equiv) resulted in decreased conversion, attributed to increased termination reactions resulting from the higher radical concentration. These results represent the lowest temperature example of a solely thermal depolymerization of an ATRP-synthesized polymer, with conversions on par with those achieved in RAFT depolymerizations under comparable conditions.^18^

To further explore the scope of the system, we investigated whether this ICAR effect could also be observed with other ligands. Changing the polymer RUC to 50 mM to aid analysis and demonstrate that higher concentrations are also possible, we conducted depolymerizations with varying amounts of ABCN in the presence of Cu^II^Cl_2_ and common ligands with differing activity:^31^ Tris(2-(dimethylamino)ethyl)amine (Me_6_Tren), TPMA, *N*,*N*,*N*′,*N*′,*N*″-pentamethyldiethylenetriamine (PMDETA), and 1,1,4,7,10,10-hexamethyltriethylenetetramine (HMTETA). All ligands tested showed negligible depolymerization in the absence of initiator (Figure 2D, Tables S4–5), and a sharp increase in conversions when ABCN was present, typically attaining ∼70% depolymerization with 0.5 or 1 equiv of ABCN. Such conversions are in agreement with previous reports of both ATRP and RAFT synthesized PBzMA at the higher 50 mM RUC.^18,26^ The broad ligand compatibility extends the versatility and applicability of the system and also highlights the potential for using more cost-effective and readily available ligands, such as PMDETA^32,33^ at lower temperatures.

The kinetics of the depolymerization process under optimized conditions with PMDETA were assessed by performing a parallel series of batch reactions and removing individual test tubes at regular intervals. Depolymerization reaches a maximum of 70% conversion (77% depolymerization efficiency) between 50 and 60 min (Figures 2E, Table S6). This reaction time is significantly shorter than previously reported depolymerizations of RAFT-synthesized poly(methacrylates) (∼8 h)^18^ while achieving similar monomer yields. The ability to conduct ICAR depolymerization at 120 °C in a 1 h time frame makes reaction monitoring more user-friendly while also using significantly less energy than at 170 °C. Taking Arrhenius parameters for decomposition from the literature, it is possible to estimate the rate at which ABCN is consumed during the depolymerization process (Table S1).^34^ The blue trace in Figure 2E shows how much ABCN is expected to remain as the depolymerization proceeds, where >99% has decomposed after 60 min, similar to the time taken to achieve maximum conversion. Adding a second aliquot of initiator to the depolymerization after 60 min had no further effect on conversion, and the maximum conversion obtained was already comparable to optimized conditions previously reported for both RAFT and ATRP type depolymerizations at similar temperatures and concentrations.^18,26^

Iron complexes have previously been employed in successful depolymerizations of ATRP-synthesized polymers^35^ and are of particular interest due to their lower toxicity and cost.^36,37^ We were therefore interested in whether ICAR depolymerization could also be applied to iron catalysts. Attempting depolymerization with Fe^III^Cl_3_ and tetrabutylammonium bromide (TBABr; 50 mM RUC, Figure 3A, Table S7) resulted in just 12% conversion at 120 °C. Addition of initiator significantly increased depolymerization conversion (up to 68% with 1 equiv of ABCN) by generating Fe^II^ species, with high initiator concentrations giving lower conversions due to excessive termination (Figure 3B), thus demonstrating that ICAR depolymerization can be applied to both Cu and Fe systems. The kinetics of iron-mediated depolymerization were also found to be similar to the copper-mediated system, with maximum conversion reached between 50 and 60 min (Figure 3C, Table S8).

**Figure 3:**
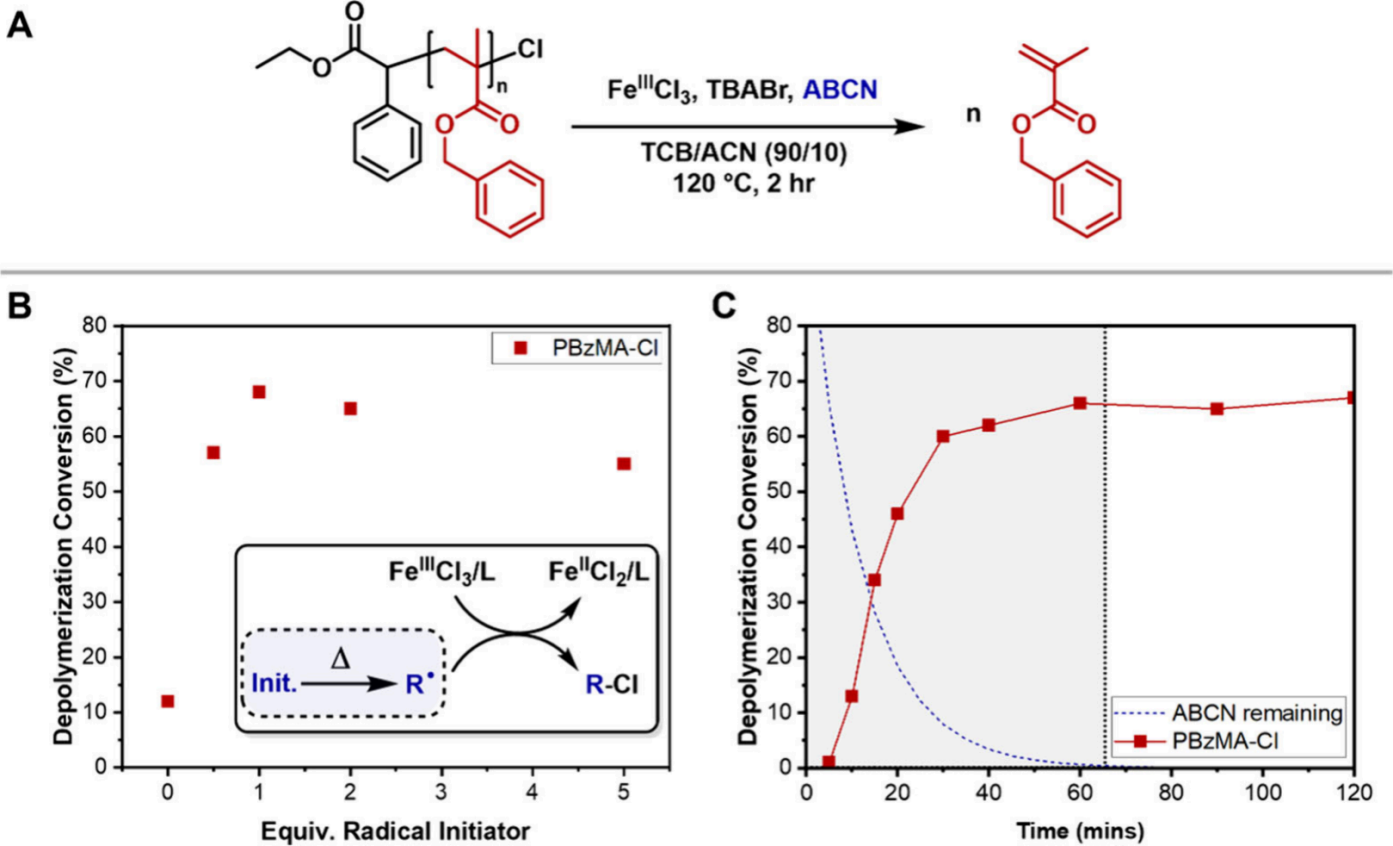
(A) ICAR depolymerization in the presence of an iron catalyst. (B) Optimization of depolymerization of PBzMA (50 mM RUC) with Fe^III^Cl_3_/TBABr and ABCN and proposed mechanism of Fe^II^ formation. (C) Kinetics of depolymerization (PBzMA, 50 mM, Fe^III^Cl_3_/TBABr, 1 equiv of ABCN).

One of the main disadvantages of carrying out depolymerization of ATRP-synthesized polymers at 170 °C is lactonization, a side reaction (Scheme S10) causing loss of end-group fidelity, yielding polymer chains that cannot be activated and depropagate to give monomer.^22,38^ As the ICAR approach allows us to achieve depolymerization at a much lower temperature, we investigated whether end-group loss was less prevalent under our conditions by incubating the polymer prior to catalyst addition (Figure 4A, Table S9). We first heated a solution of PBzMA at 120 °C for 2 h, then added Cu^II^Cl_2_/PMDETA and ABCN and heated for a further hour. As expected, no monomer was detected by ^1^H NMR spectroscopy after 2 h. However, sampling after the catalyst addition and further heating gave a depolymerization conversion of 70%, identical to a control reaction, where the catalyst was added from the start of the reaction, leading to the conclusion that lactonization is not significant under our conditions. Previous studies of ATRP polymers incubated at 170 °C have shown markedly lower depolymerization conversions as a result of lactonization, highlighting the benefit of ICAR in suppressing side reactions.^26^ The high end-group retention of ICAR depolymerization at 120 °C even persists in the presence of Cu^II^Cl_2_/PMDETA; allowing for “on-demand” depolymerization where significant monomer generation only commences upon ABCN addition, yielding 70% monomer after a further hour of reaction time (Table S9).

**Figure 4:**
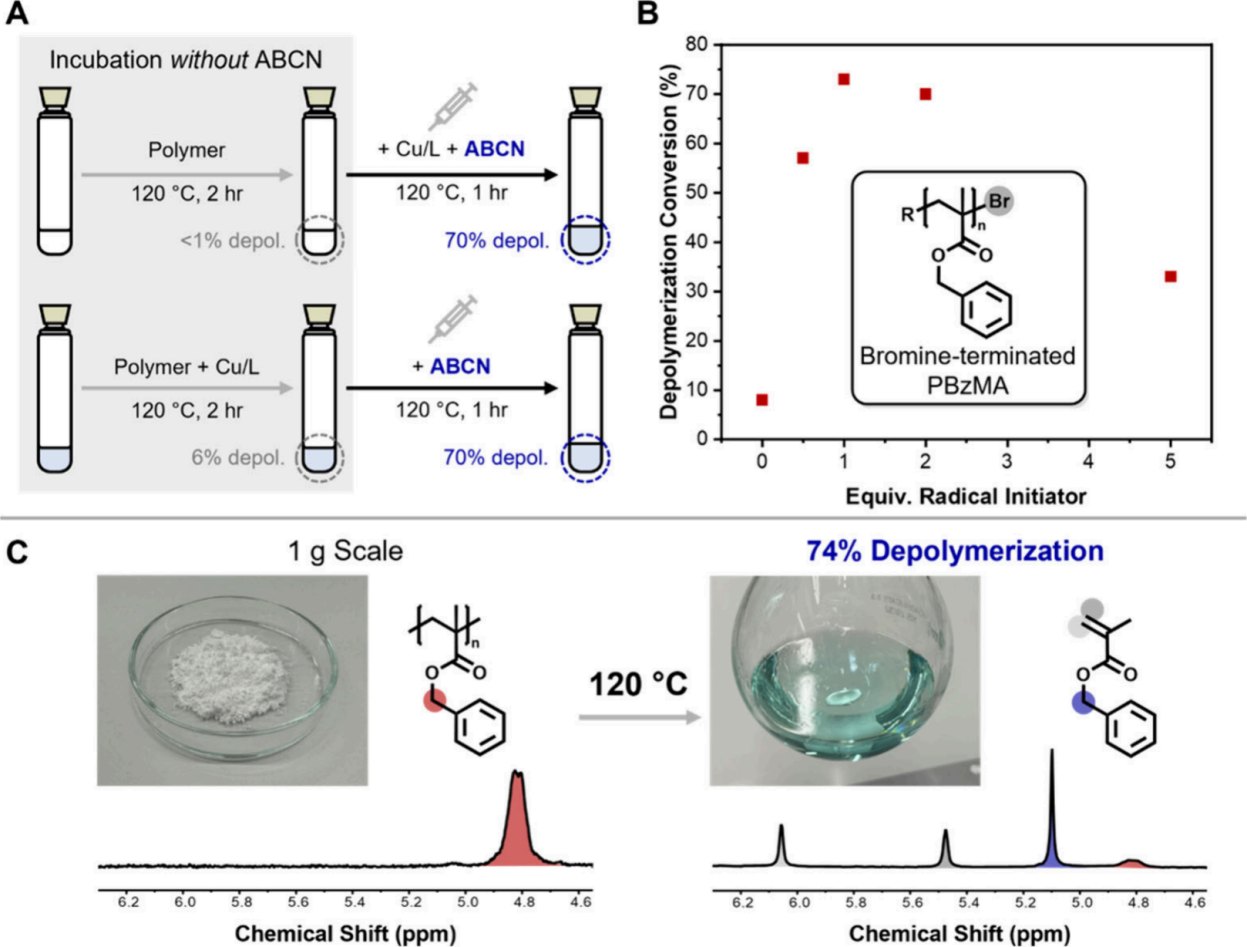
(A) Incubation experiments to demonstrate retention of end-group fidelity. (B) Optimization of depolymerization of PBzMA-Br (50 mM RUC) with Cu^II^Cl_2_/PMDETA and ABCN. (C) 1 g scale depolymerization of PBzMA (50 mM, Cu^II^Cl_2_/PMDETA, 1 equiv of ABCN) in a round-bottom flask.

Encouraged by the end-group retention observed for PBzMA-Cl at 120 °C, we attempted the same reactions with PBzMA-Br, synthesized by photoinduced iron-catalyzed ATRP (Figure S1–2, Table S2). Although bromine terminated polymers are common in ATRP, Br is a better leaving group than Cl and is thus more susceptible to lactonization. The one previous report of their depolymerization was achieved by maximizing depolymerization rate at 170 °C to outcompete lactonization, although Br-terminated polymers still yield lower percentages of monomer recovery compared to Cl-terminated analogues under otherwise identical conditions.^25^ ICAR depolymerization utilizing a Cu^II^Cl_2_/PMDETA catalyst system with ABCN yielded up to 73% depolymerization at 120 °C (Figure 4B, Table S10). It is noted that this is the first report whereby both Br- and Cl-terminated polymers result in identical depolymerization yields, further demonstrating the broad scope that ICAR depolymerization offers.

As the ICAR process relies on heat and a chemical stimulus (radical initiator) to generate active species, it could offer more scale-up potential than previously reported photothermal approaches, which require both heat and light. To test this, we conducted a 50 mM RUC depolymerization of 1 g of PBzMA-Cl under optimized conditions (120 °C, Cu^II^Cl_2_/PMDETA, 1 equiv. ABCN, ∼114 mL total solution volume), which yielded 74% monomer regeneration in 1 h (Figure 4C); the highest scale solution depolymerization of an RDRP-synthesized polymer reported to date.

In summary, ICAR depolymerization is shown to be an effective and generally applicable method of reducing the required depolymerization temperatures by 50 °C for a wide range of catalysts encompassing both copper and iron systems. By simply adding a small amount of a commercially available radical initiator, such as ABCN, the depolymerization efficiency could reach near-quantitative levels (up to 96%), resulting in the regeneration of high amounts of pristine monomer. Under our judiciously optimized reaction conditions, side reactions including lactonization were significantly minimized, leading to the retention of end-group fidelity at the lower temperatures afforded by ICAR depolymerization. Thanks to these reduced side reactions, both chlorine and bromine terminated polymers could be effectively depolymerized.

## Supporting Information Available

Experimental details, materials, methods, ^1^H NMR and SEC spectra, and tables of depolymerization conversions (PDF)
