# Supplementary material for: Initiators for Continuous Activator Regeneration (ICAR) Depolymerization
Source: J Am Chem Soc. 2024 Dec 12;146(51):35023–8. doi: 10.1021/jacs.4c13785 (PMC11673573; doi:10.1021/jacs.4c13785)
Supplement: Supplementary file 1 — ja4c13785_si_001.pdf [file ja4c13785_si_001.pdf]

Supporting Information

Initiators for Continuous Activator Regeneration (ICAR) Depolymerization

Glen R. Jones,[a] Maria-Nefeli Antonopoulou,[a] Nghia P. Truong,[a] Athina Anastasaki*[a]

[a] Laboratory for Polymeric Materials, Department of Materials, ETH Zürich, Vladimir-Prelog-Weg 5, 8093 Zürich, Switzerland

*Athina Anastasaki: athina.anastasaki@mat.ethz.ch

Materials

All chemicals were purchased from Sigma-Aldrich and used without additional purification unless otherwise stated. Benzyl methacrylate (>98.0%) was purchased from Tokyo Chemical Industry (TCI) and was filtered through basic alumina to remove inhibitor prior to use. 1,2,4-trichlorobenzene and Ethyl α-chlorophenyl acetate (97%) were purchased from Acros Organics. Tris-(2-(dimethylamino)ethyl)amine (Me_6Tren) was synthesized according to literature procedure[1] and was distilled under vacuum prior to use.

Instrumentation

^1H NMR spectra were collected on Bruker DPX-300 or DPX-400 spectrometers with deuterated chloroform ($CDCl_3$) or deuterated acetone (acetone-d_6). Monomer conversions for polymerizations and depolymerizations were determined by comparison of integrals of vinylic protons to methylene protons from both monomer and polymer. SEC analysis was performed using a modular Shimadzu system comprising of a CBM-20A system controller, an SIL-20A automatic injector, a 10.0 µm bead-size guard column (50 × 7.5 mm) followed by three KF-805L columns (300 × 8 mm, bead size: 10 µm, pore size maximum: 5000 Å), an SPD-20A ultraviolet detector, and an RID-20A differential refractive-index detector. Column temperature was maintained at 40 °C using a CTO-2A oven. *N,N*-dimethylacetamide (DMAc, HPLC grade) was used as the eluent with 0.03% w/v LiBr. Calibration was performed using commercial narrow molar mass distribution poly(methyl methacrylate) (PMMA) standards with molar masses ranging from 5×10^3 to 1.5×10^6. SEC samples were dissolved in DMAc and filtered through a short column of basic alumina to remove residual metals.

Experimental Procedures

General procedure for synthesis of chlorine terminated poly(benzyl methacrylate) by ARGET-ATRP

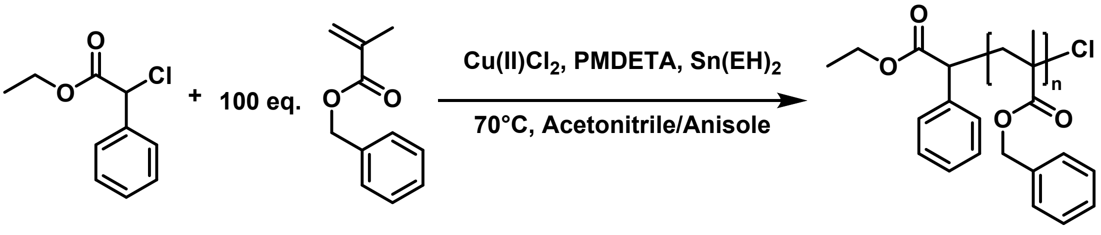

Scheme S1: ARGET-ATRP of benzyl methacrylate. [BzMA] : [ECPA] : [$CuCl_2$] : [PMDETA] : [$Sn(EH)_2$)] = [100] : [1] : [0.15] : [0.2] : [0.08]. Polymerization was conducted in 95/5 mixture of acetonitrile and anisole with 50 vol% monomer.

A round bottom flask was charged with 23.8 mg of $CuCl_2$ (0.177 mmol, 0.15 equiv.) and a stirrer bar. 19 mL acetonitrile was added with 49.3 µL *N,N,N',N'',N''*-pentamethyldiethylenetriamine (PMDETA, 0.236 mmol, 0.2 equiv.). The reaction vessel was briefly sonicated in a benchtop sonication bath to aid solubilization, resulting in a pale blue solution. 20 mL benzyl methacrylate (118 mmol, 100 equiv.) and 20.3 µL ethyl α-chlorophenyl acetate (ECPA, 1.18 mmol, 1 equiv.) was then added and the flask was fitted with a rubber septum and deoxygenated by nitrogen sparging for 20 minutes. Concurrently, a 0.095 mM solution of tin(II) 2-ethylhexanoate ($Sn(EH)_2$) in anisole was prepared, charged to a 5 mL glass vial, sealed with a rubber septum, and deoxygenated via nitrogen sparging for 20 minutes. After deoxygenation, 1 mL of the tin solution (0.094 mmol, 0.08 equiv.) was transferred to the round bottom flask with a deoxygenated syringe, resulting in the solution turning a deep blue. The round bottom flask was then immersed in an oil bath at 70 °C with magnetic stirring (360 rpm). After 4 hours the reaction was stopped by removing the round bottom flask from the oil bath and exposing the solution to air. Aliquots were taken for ^{1}H NMR and SEC analysis. The remaining polymerization solution was then diluted with acetone and passed through a column of basic alumina to remove the catalyst. The resulting solution was concentrated *in vacuo* and precipitated into cold methanol and dried in a vacuum oven. The purified PBzMA-Cl was analyzed via ^{1}H NMR to confirm all trace monomer had been removed, and SEC to measure molar mass and dispersity.

Chain extension of poly(benzyl methacrylate)-Cl with BzMA by ARGET-ATRP

A stock solution of $Cu^{II}Cl_2$ and PMDETA was first prepared by charging 1.0 mg of $Cu^{II}Cl_2$ (7.4×10^{-3} mmol) and 5.1 µL PMDETA (2.44×10^{-2} mmol) to a 5 mL glass vial, adding 0.15 mL of ACN and 2.85 mL anisole and briefly sonicating to ensure dissolution of the catalyst. A 5 mL glass vial was charged with a stirrer bar, 100 mg of PBzMA-Cl (polymer 1), 0.5 mL of catalyst stock solution, and 370 µL of BzMA (200 eq. with respect to polymer chains, 2.2 mmol), sealed with a septum and deoxygenated by nitrogen sparging for 20 minutes. Concurrently, a $Sn(EH_2)$ stock solution was prepared by dissolving 39 mg of $Sn(EH_2)$ (9.6×10^{-2} mmol) in 2.2 mL of anisole, which was also deoxygenated for 20 minutes. 50 µL of the tin stock solution was transferred to the reaction vial via deoxygenated syringe and the reaction was heated at 70 °C in an oil bath (360 rpm stirring) for 3.5 hours. After which time the reaction was sampled and analyzed by ^{1}H NMR and SEC.

Synthesis of bromine terminated poly(benzyl methacrylate) by photoinduced ATRP

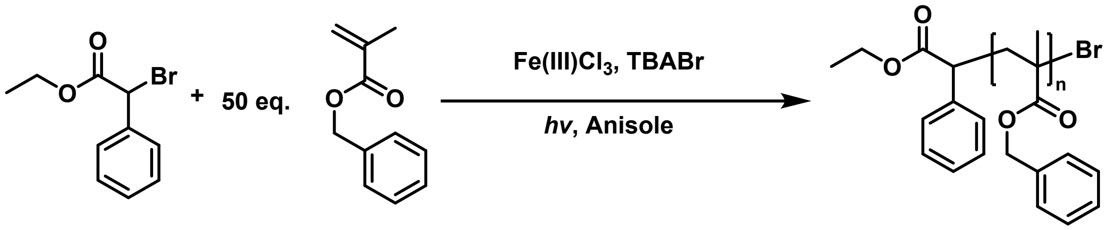

Scheme S2: Photoinduced ATRP of benzyl methacrylate. [BzMA] : [EBPA] : [$FeCl_3$] : [TBABr] = [50] : [1] : [0.1] : [0.1]. Polymerization was conducted in anisole with 50 vol% monomer.

A 30 mL glass vial was charged with 17.4 mg of $FeBr_3$ (0.059 mmol, 0.1 equiv.) and a stirrer bar. 5 mL anisole was added with 19.0 mg of tetrabutylammoniumbromide (TBABr, 0.059 mmol, 0.1 equiv.). The reaction vessel was briefly sonicated in a benchtop sonication bath to aid solubilization, resulting in a deep orange solution. 5 mL benzyl methacrylate (30 mmol, 50 equiv.) and 103.3 µL ethyl α-bromophenyl acetate (EBPA, 0.590 mmol, 1 equiv.) was then added and the flask was fitted with a rubber septum and deoxygenated by nitrogen sparging for 20 minutes. The vial was then placed onto a magnetic stirrer plate and irradiated with blue LED lights (48 W, λ ~ 465 nm) for 1.5 hours. The reaction was stopped by ceasing irradiation and exposing the solution to air. Aliquots were taken for ^{1}H NMR and SEC analysis. The remaining polymerization solution was then diluted with acetone and passed through a column of basic alumina to remove the catalyst. The resulting solution was concentrated *in vacuo* and precipitated into cold methanol and dried in a vacuum oven. The purified PBzMA-Br was

analyzed via ^{1}H NMR to confirm all trace monomer had been removed, and SEC to measure molar mass and dispersity.

General procedure for ICAR depolymerization of PBzMA-Cl (5 mM RUC) with $Cu^{II}Cl_2$/TPMA (1 equiv. ABCN)

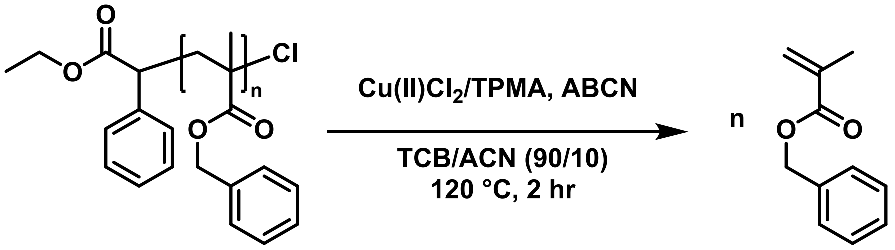

Scheme S3: ICAR depolymerization of PBzMA-Cl. [PBzMA-Cl] : [$CuCl_2$] : [TPMA] : [ABCN] = [1] : [1] : [1] : [1].

Depolymerization was carried out with 1 equiv. of catalyst with respect to PBzMA-Cl. A stock solution of $Cu^{II}Cl_2$ and TPMA was first prepared by charging 3.9 mg of $Cu^{II}Cl_2$ (0.029 mmol) and 8.3 mg tris(2-pyridylmethyl)amine (TPMA, 0.029 mmol) to a 5 mL glass vial, 3 mL of acetonitrile (ACN) was added and the solution was briefly sonicated to ensure dissolution of the catalyst. Stock solutions of PBzMA in 1,2,4-trichlorobenzene (TCB) (1.76 mg/ml), and 1,1′-Azobis(cyclohexanecarbonitrile) (ABCN, 2.92 mg/mL) in TCB were also prepared in a similar fashion. For a typical depolymerization, a 10 mL glass test tube was charged with 10 μL of catalyst stock solution (0.013 mg $Cu^{II}Cl_2$, 9.6×10^{-5} mmol, 0.028 mg TPMA, , 9.6×10^{-5} mmol) and 90 μL of ACN. 500 μL PBzMA-Cl stock solution (0.88 mg PBzMA-Cl, 9.6×10^{-5} mmol), 392 μL of TCB and 8 μL of ABCN stock solution (0.024 mg ABCN, 9.6×10^{-5} mmol) were then added and the test tube was sealed with a rubber septum and secured with a cable tie. The depolymerization solution was then deoxygenated by nitrogen sparging for 10 minutes before being placed in an oil bath at 120 °C. After 2 hours the test tube was removed from the oil bath, placed into an ice bath and opened to air. Depolymerization conversion was determined by ^1H NMR in $CDCl_3$. ABCN concentrations were varied between experiments by changing the ratio between the amounts of TCB and ABCN stock solution added.

General procedure for ICAR depolymerization of PBzMA-Cl (50 mM RUC) with $Cu^{II}Cl_2$ and PMDETA (1 equiv. ABCN)

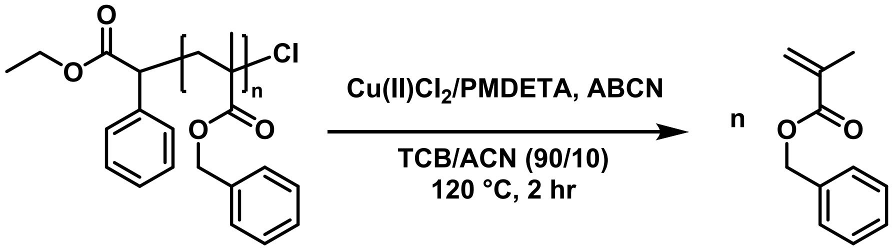

Scheme S4: ICAR depolymerization of PBzMA-Cl. [PBzMA-Cl] : [$CuCl_2$] : [PMDETA] : [ABCN] = [1] : [1] : [1] : [1].

Depolymerization was carried out with 1 equiv. of catalyst with respect to PBzMA-Cl. A stock solution of $Cu^{II}Cl_2$ and PMDETA was first prepared by charging 3.9 mg of $Cu^{II}Cl_2$ (0.029 mmol) to a 5 mL glass vial, adding 3 mL of ACN and 6.0 μL PMDETA (0.029 mmol) and briefly sonicating to ensure dissolution of the catalyst. Stock solutions of PBzMA-Cl in TCB (17.6 mg/ml), and ABCN (2.92 mg/mL) in TCB were also prepared in a similar fashion. For a typical depolymerization, a 10 mL glass test tube was charged with 100 μL of catalyst stock solution (0.13 mg $Cu^{II}Cl_2$, 9.6×10^{-4} mmol, 0.20 μL PMDETA, , 9.6×10^{-4} mmol). 500 μL PBzMA-Cl stock solution (8.8 mg PBzMA-Cl, 9.6×10^{-4} mmol), 320 μL of TCB and 80 μL of ABCN stock solution (0.24 mg ABCN, 9.6×10^{-4} mmol) were then added and the test tube was sealed with a rubber septum and secured with a cable tie. The depolymerization solution was then deoxygenated by nitrogen sparging for 10 minutes before being placed in an oil bath at 120 °C. After 2 hours the test tube was removed from the oil bath, placed into an ice bath and opened to air. Depolymerization conversion was determined by ^1H NMR in $CDCl_3$. ABCN concentrations were varied between experiments by changing the ratio between the amounts of TCB and ABCN stock solution added.

General procedure for depolymerization kinetics

Depolymerization kinetics were performed by preparing multiple identical reactions from the same stock solutions, as described above. Reactions were deoxygenated in parallel and placed within the same oil bath at 120 °C. Individual test tubes were taken out of the oil bath at regular intervals and immediately exposed to air and placed in an ice bath to cease polymerization.

Decomposition of ABCN at 120 °C

In order to estimate the decomposition rate of ABCN at 120 °C, Arrhenius parameters were taken from the literature (table S1),[2] yielding a k_d value of 0.00141 s^{-1} at 120 °C. The amount of initiator remaining in a depolymerization reaction as a function of time was estimated by using a simplified first order rate equation.

Table S1: Arrhenius parameters for ABCN decomposition.

A (s^{-1})	E_a (KJ/mol)	$k_{d\ 120℃}$ (s^{-1})
1.1×10^{16}	142.2	0.00141

General procedure for ICAR depolymerization of PBzMA-Cl (50 mM RUC) with $Fe^{III}Cl_3$/TBABr

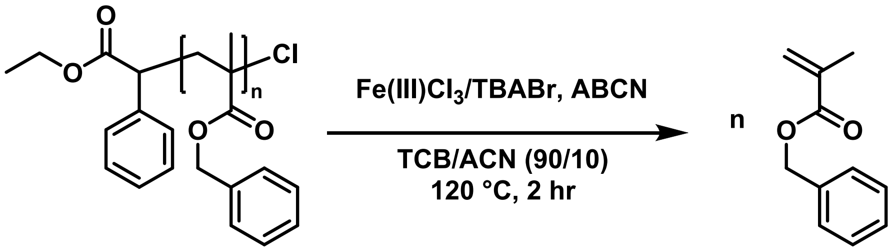

Scheme S5: ICAR depolymerization of PBzMA-Cl. [PBzMA-Cl] : [$FeCl_3$] : [TBABr] : [ABCN] = [1] : [1] : [1] : [1].

Depolymerization was carried out with 1 equiv. of catalyst with respect to PBzMA-Cl. A stock solution of $Fe^{III}Cl_3$ and tetrabutylammoniumbromide (TBABr) was first prepared by charging 4.7 mg of $Fe^{III}Cl_3$ (0.029 mmol) and 11.1 mg TBABr (0.029 mmol) to a 5 mL glass vial, 3 mL of ACN was added and the solution was briefly sonicated to ensure dissolution of the catalyst. Stock solutions of PBzMA-Cl in TCB (17.6 mg/ml), and ABCN (2.92 mg/mL) in TCB were also prepared in a similar fashion. For a typical depolymerization, a 10 mL glass test tube was charged with 100 μL of catalyst stock solution (0.16 mg $Fe^{III}Cl_3$, 9.6×10^{-4} mmol, 0.37 mg TBABr, , 9.6×10^{-4} mmol). 500 μL PBzMA-Cl stock solution (8.8 mg PBzMA-Cl, 9.6×10^{-4} mmol), 320 μL of TCB and 80 μL of ABCN stock solution (0.24 mg ABCN, 9.6×10^{-4} mmol) were then added and the test tube was sealed with a rubber septum and secured with a cable tie. The depolymerization solution was then deoxygenated by nitrogen sparging for 10 minutes before being placed in an oil bath at 120 °C. After 2 hours the test tube was removed from the oil bath, placed into an ice bath and opened to air. Depolymerization conversion was determined by ^1H NMR in $CDCl_3$. ABCN concentrations were varied between experiments by changing the ratio between the amounts of TCB and ABCN stock solution added.

Incubation of PBzMA-Cl prior to depolymerization

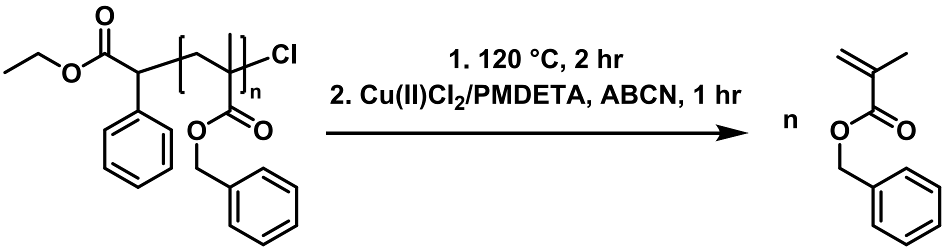

Scheme S6: Incubation of PBzMA-Cl prior to catalyst and initiator addition. [PBzMA-Cl] : [$CuCl_2$] : [PMDETA] : [ABCN] = [1] : [1] : [1] : [1].

Two test tubes were each charged with 500 µL of PBzMA-Cl stock solution (8.8 mg PBzMA-Cl, 9.6×10^{-4} mmol) and 320 µL of TCB before being sealed with a rubber septum and deoxygenated by sparging with nitrogen for 10 minutes. The test tubes were then placed in an oil bath at 120 °C for 2 hours. After this time 1 test tube was removed, placed into an ice bath and exposed to air. 80 µL of ABCN stock solution (0.24 mg ABCN, 9.6×10^{-4} mmol) and 100 µL of catalyst stock solution (0.13 mg $Cu^{II}Cl_2$, 9.6×10^{-4} mmol, 0.20 µL PMDETA, , 9.6×10^{-4} mmol) which had been deoxygenated by nitrogen sparging were injected into the second test tube and left to react at 120°C for a further hour. Conversion was determined by ^{1}H NMR in $CDCl_3$.

Incubation of PBzMA-Cl with $Cu^{II}Cl_2$/PMDETA prior to initiator addition

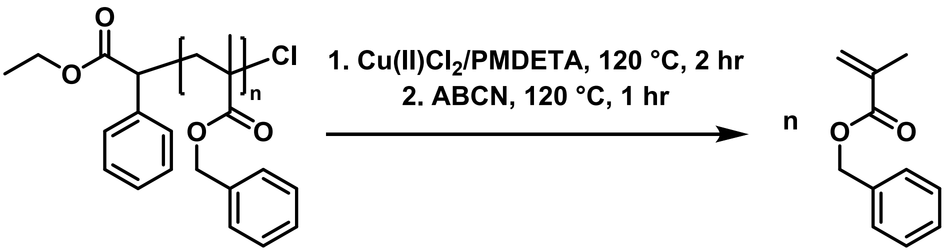

Scheme S7: Incubation of PBzMA-Cl with catalyst prior to initiator addition. [PBzMA-Cl] : [$CuCl_2$] : [PMDETA] : [ABCN] = [1] : [1] : [1] : [1].

Two test tubes were each charged with 500 µL of PBzMA-Cl stock solution (8.8 mg PBzMA-Cl, 9.6×10^{-4} mmol), 100 µL of catalyst stock solution (0.13 mg $Cu^{II}Cl_2$, 9.6×10^{-4} mmol, 0.20 µL PMDETA, , 9.6×10^{-4} mmol), and 320 µL of TCB before being sealed with a rubber septum and deoxygenated by sparging with nitrogen for 10 minutes. The test tubes were then placed in an oil bath at 120 °C for 2

hours. After this time 1 test tube was removed, placed into an ice bath and exposed to air. 80 μL of ABCN stock solution (0.24 mg ABCN, 9.6×10^{-4} mmol) and which had been deoxygenated by nitrogen sparging were injected into the second test tube and left to react at 120°C for a further hour. Conversion was determined by ^{1}H NMR in $CDCl_3$.

Example ICAR depolymerization of PBzMA-Br (50 mM RUC) with $Cu^{II}Cl_2$/PMDETA

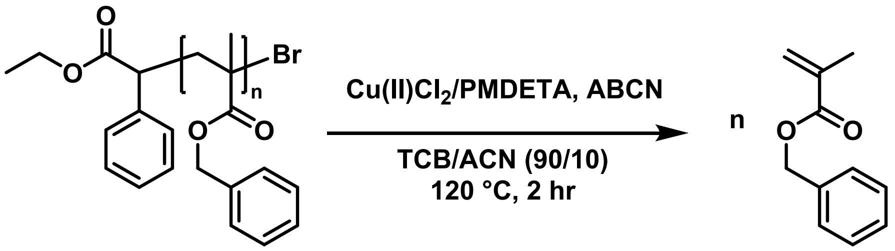

Scheme S8: ICAR depolymerization of PBzMA-Br. [PBzMA-Br] : [$CuCl_2$] : [PMDETA] : [ABCN] = [1] : [1] : [1] : [1].

Depolymerization was carried out with 1 equiv. of catalyst with respect to PBzMA-Br. A stock solution of $Cu^{II}Cl_2$ and PMDETA was first prepared by charging 7.1 mg of $Cu^{II}Cl_2$ (0.052 mmol) to a 5 mL glass vial, adding 3 mL of ACN and 10.9 µL PMDETA (0.052 mmol) and briefly sonicating to ensure dissolution of the catalyst. Stock solutions of PBzMA-Br in TCB (17.6 mg/ml), and ABCN (5.4 mg/mL) in TCB were also prepared in a similar fashion. For a typical depolymerization, a 10 mL glass test tube was charged with 100 µL of catalyst stock solution (0.24 mg $Cu^{II}Cl_2$, 1.8×10^{-3} mmol, 0.36 µL PMDETA, , 1.8×10^{-3} mmol). 500 µL PBzMA-Br stock solution (8.8 mg PBzMA-Br, 1.8×10^{-3} mmol), 320 µL of TCB and 80 µL of ABCN stock solution (0.43 mg ABCN, 1.8×10^{-3} mmol) were then added and the test tube was sealed with a rubber septum and secured with a cable tie. The depolymerization solution was then deoxygenated by nitrogen sparging for 10 minutes before being placed in an oil bath at 120 °C. After 2 hours the test tube was removed from the oil bath, placed into an ice bath and opened to air. Depolymerization conversion was determined by ^{1}H NMR in $CDCl_3$. ABCN concentrations were varied between experiments by changing the ratio between the amounts of TCB and ABCN stock solution added.

Gram scale ICAR depolymerization of PBzMA-Cl (50 mM RUC) with $Cu^{II}Cl_2$ and PMDETA (1 equiv. ABCN)

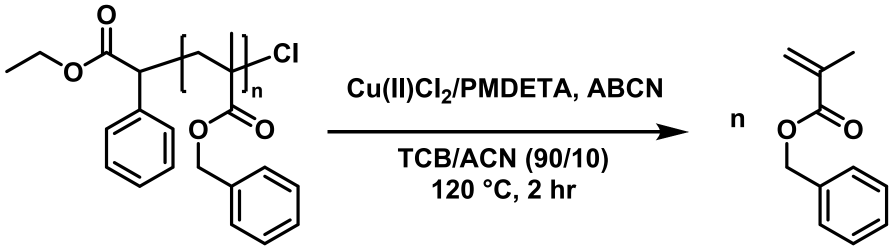

Scheme S9: ICAR depolymerization of PBzMA-Cl. [PBzMA-Cl] : [$CuCl_2$] : [PMDETA] : [ABCN] = [1] : [1] : [1] : [1].

Depolymerization was carried out with 1 equiv. of catalyst with respect to PBzMA-Cl. A 500 mL round bottom flask was charged with 1000 mg of PBzMA-Cl (0.13mmol) and 32.1 mg of ABCN (0.13 mmol) which were then dissolved in 102.3 mL of TCB. In a 30 mL glass vial, 17.6 mg $Cu^{II}Cl_2$ (0.13 mmol) was dissolved in 11.4 mL ACN and 27.4 µL of PMDETA (0.13 mmol) were added and the solution sonicated briefly. The catalyst solution was then transferred to the round bottom flask, which was sealed with a septum and deoxygenated by nitrogen sparging for 20 minutes. After this time the reaction was placed in an oil bath at 120 °C for 2 hours. After 2 hours reaction was removed from the oil bath, placed into an ice bath and opened to air. Depolymerization conversion was determined by ^1H NMR in $CDCl_3$ (74%).

Supplementary Tables and Figures

Table S2: Polymers used in this study. Polymers 1 and 3 were prepared according to the ARGET-ATRP procedure. Polymer 2 was prepared using the photoinduced ATRP procedure . [a] Used in all studies of depolymerization on a small scale. [b] Used in depolymerization studies of bromine terminated polymer. [c] Used in large scale depolymerization.

#	Polymer	Conv.	M_n (SEC), purif.	*Đ*
1[a]	PBzMA-Cl	58%	9200	1.13
2[b]	PBzMA-Br	64%	5000	1.12
3[c]	PBzMA-Cl	40%	7600	1.15

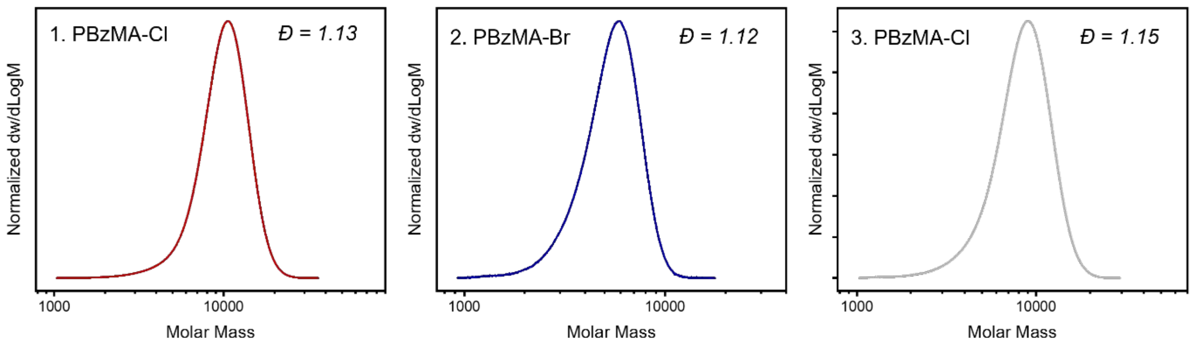

Figure S1: SEC traces of purified polymers used in this study.

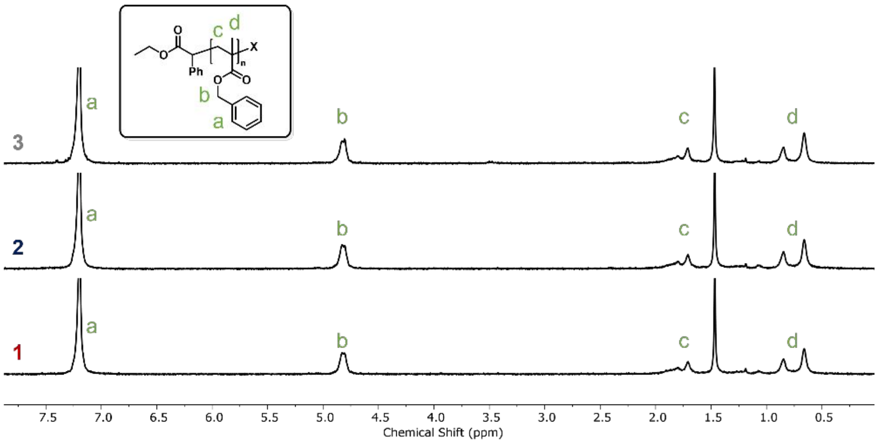

Figure S2: ^{1}H NMR spectra of purified polymers used in this study.

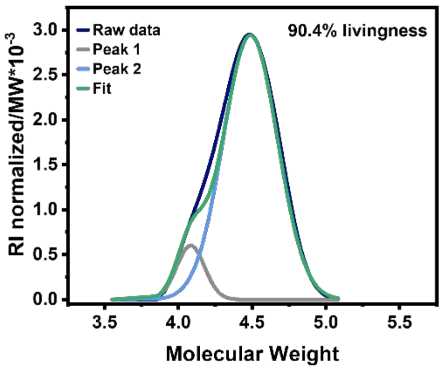

Figure S3: Deconvoluted SEC traces of the chain extended PBzMA-Br (polymer 1) to quantify livingness. Livingness was calculated by comparison of the integration area of P(BzMA-b-BzMA)-Br versus the unreacted PBzMA-Br, based on the methodology reported by Wooley and coworkers.[3]

Table S3: Depolymerization of PBzMA-Cl (5 mM RUC) in 90/10 mixture of TCB and ACN using $CuCl_2$/TPMA (1 eq. with respect to polymer end-group) and differing amounts of ABCN at 120 °C. [a] Determined by ^1H NMR.

$[CuCl_2]$: [TPMA]	[ABCN]	Time (hrs)	Conversion (%)[a]
1 : 1	0	2	<1
1 : 1	0.5	2	70
1 : 1	1	2	87
1 : 1	2	2	84
1 : 1	5	2	74
0 : 0	0	2	<1
0 : 0	0.5	2	<1
0 : 0	1	2	<1
0 : 0	2	2	<1
0 : 0	5	2	<1

Table S4: Depolymerization of PBzMA-Cl (50 mM RUC) in 90/10 mixture of TCB and ACN using $CuCl_2$/PMDETA (1 eq. with respect to polymer end-group) and differing amounts of ABCN at 120 °C. [a] Determined by ^1H NMR. [b] Determined by SEC.

[ABCN]	Conversion (%)[a]
0	3
0.5	54
1	69
2	61
5	42

Table S5: Depolymerization of PBzMA-Cl (50 mM RUC) in 90/10 mixture of TCB and ACN using $CuCl_2$ and various ligands (1 eq. with respect to polymer end-group) and differing amounts of ABCN at 120 °C. [a] Determined by ^1H NMR.

Ligand (L)	[P-Cl] : [$CuCl_2$/ [L]	[ABCN]	Time (hrs)	Conversion (%)[a]
TPMA	1 : 1	0	2	6
HMTETA	1 : 1	0	2	21
Me_6Tren	1 : 1	0	2	8
TPMA	1 : 1	0.5	2	68
HMTETA	1 : 1	0.5	2	68
Me_6Tren	1 : 1	0.5	2	68
TPMA	1 : 1	1	2	64
HMTETA	1 : 1	1	2	67
Me_6Tren	1 : 1	1	2	66
TPMA	1 : 1	2	2	56
HMTETA	1 : 1	2	2	59
Me_6Tren	1 : 1	2	2	57
TPMA	1 : 1	5	2	42
HMTETA	1 : 1	5	2	41
Me_6Tren	1 : 1	5	2	41

Table S6: Kinetics of depolymerization of PBzMA-Cl (50 mM RUC) in 90/10 mixture of TCB and ACN using $CuCl_2$/PMDETA (1 eq. with respect to polymer end-group) and 1 equiv. ABCN at 120 °C. [a] Determined by ^{1}H NMR. [b] Determined by SEC.

[ABCN]	Time (mins)	Conversion (%)[a]
1	0	0
1	5	2
1	10	4
1	15	19
1	20	48
1	30	60
1	40	65
1	60	69
1	90	70
1	120	70

Table S7: Depolymerization of PBzMA-Cl (50 mM RUC) in 90/10 mixture of TCB and ACN using $FeCl_3$/TBABr (1 eq. with respect to polymer end-group) and differing amounts of ABCN at 120 °C. [a] Determined by ^{1}H NMR.

[ABCN]	Time (hrs)	Conversion (%)[a]
0	2	12
0.5	2	57
1	2	68
2	2	65
5	2	55

Table S8: Kinetics of depolymerization of PBzMA-Cl (50 mM RUC) in 90/10 mixture of TCB and ACN using $FeCl_3$/TBABr (1 equiv. with respect to polymer end-group) and 1 equiv. ABCN at 120 °C. [a] Determined by ^{1}H NMR.

[ABCN]	Time (mins)	Conversion (%)[a]
1	0	0
1	5	1
1	10	13
1	15	34
1	20	46
1	30	60
1	40	62
1	60	66
1	90	65
1	120	67

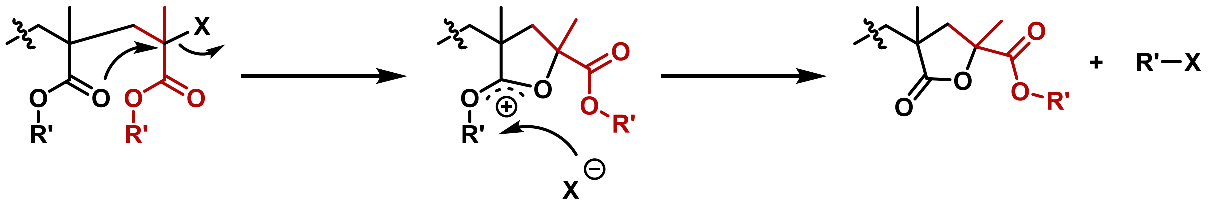

Scheme S10: Proposed mechanism of lactonization of chain-ends of ATRP polymers.[4]

Table S9: Incubation experiments prior to cat./init. addition and init. addition. Depolymerization of PBzMA-Cl (50 mM RUC) in 90/10 mixture of TCB and ACN using $CuCl_2$/PMDETA (1 eq. with respect to polymer end-group) and 1 equiv. ABCN at 120 °C. [a] Determined by ^1H NMR.

[P-Cl] : [$CuCl_2$] : [PMDETA]	[ABCN]	Time (hrs)	Conversion (%)[a]
1 : 0 : 0	0	2	0
1 : +1 : +1	+1	1 (3 total)	70
1 : 1 : 1	0	2	6
1 : 1 : 1	+1	1 (3 total)	70

Table S10: Depolymerization of PBzMA-Br (50 mM RUC) in 90/10 mixture of TCB and ACN using $CuCl_2$/PMDETA (1 eq. with respect to polymer end-group) and differing amounts of ABCN at 120 °C. [a] Determined by ^{1}H NMR.

[ABCN]	Time (hrs)	Conversion (%)[a]
0	2	8
0.5	2	57
1	2	73
2	2	70
5	2	33

References

(1) Ciampolini, M.; Nardi, N. Five-Coordinated High-Spin Complexes of Bivalent Cobalt, Nickel, andCopper with Tris (2-dimethylaminoethyl) amine. *Inorg. Chem.* **1966**, *5* (1), 41-44.

(2) Anžlovar, A.; Orel, Z. C.; Žigon, M. Poly (methyl methacrylate) composites prepared by in situ polymerization using organophillic nano-to-submicrometer zinc oxide particles. *Eur. Polym. J.* **2010**, *46* (6), 1216-1224.

(3) Bartels, J. W.; Cauët, S. I.; Billings, P. L.; Lin, L. Y.; Zhu, J.; Fidge, C.; Pochan, D. J.; Wooley, K. L. Evaluation of isoprene chain extension from PEO macromolecular chain transfer agents for the preparation of dual, invertible block copolymer nanoassemblies. *Macromolecules* **2010**, *43* (17), 7128-7138.

(4) Martinez, M. R.; De Luca Bossa, F.; Olszewski, M.; Matyjaszewski, K. Copper (II) Chloride/Tris (2-pyridylmethyl) amine-Catalyzed Depolymerization of Poly (n-butyl methacrylate). *Macromolecules* **2021**.
